# ENIGMA and the individual: Predicting factors that affect the brain in 35 countries worldwide^☆☆☆★^

**DOI:** 10.1016/j.neuroimage.2015.11.057

**Published:** 2015-12-04

**Authors:** Paul M. Thompson, Ole A. Andreassen, Alejandro Arias-Vasquez, Carrie E. Bearden, Premika S. Boedhoe, Rachel M. Brouwer, Randy L. Buckner, Jan K. Buitelaar, Kazima B. Bulayeva, Dara M. Cannon, Ronald A. Cohen, Patricia J. Conrod, Anders M. Dale, Ian J. Deary, Emily L. Dennis, Marcel A. de Reus, Sylvane Desrivieres, Danai Dima, Gary Donohoe, Simon E. Fisher, Jean-Paul Fouche, Clyde Francks, Sophia Frangou, Barbara Franke, Habib Ganjgahi, Hugh Garavan, David C. Glahn, Hans J. Grabe, Tulio Guadalupe, Boris A. Gutman, Ryota Hashimoto, Derrek P. Hibar, Dominic Holland, Martine Hoogman, Hilleke Hulshoff Pol, Norbert Hosten, Neda Jahanshad, Sinead Kelly, Peter Kochunov, William S. Kremen, Phil H. Lee, Scott Mackey, Nicholas G. Martin, Bernard Mazoyer, Colm McDonald, Sarah E. Medland, Rajendra A. Morey, Thomas E. Nichols, Tomas Paus, Zdenka Pausova, Lianne Schmaal, Gunter Schumann, Li Shen, Sanjay M. Sisodiya, Dirk J.A. Smit, Jordan W. Smoller, Dan J. Stein, Jason L. Stein, Roberto Toro, Jessica A. Turner, Martijn P. van den Heuvel, Odile L. van den Heuvel, Theo G.M. van Erp, Daan van Rooij, Dick J. Veltman, Henrik Walter, Yalin Wang, Joanna M. Wardlaw, Christopher D. Whelan, Margaret J. Wright, Jieping Ye, the ENIGMA Consortium

**Affiliations:** a Imaging Genetics Center, Mark and Mary Stevens Institute for Neuroimaging & Informatics, Keck School of Medicine of the University of Southern California, Marina del Rey 90292, USA; b NORMENT-KG Jebsen Centre, Institute of Clinical Medicine, University of Oslo, Oslo 0315, Norway; c NORMENT-KG Jebsen Centre, Division of Mental Health and Addiction, Oslo University Hospital, Oslo 0315, Norway; d Donders Center for Cognitive Neuroscience, Departments of Psychiatry, Human Genetics & Cognitive Neuroscience, Radboud University Medical Center, Nijmegen 6525, The Netherlands; e Department of Psychiatry & Biobehavioral Sciences, University of California, Los Angeles, CA 90095, USA; f Dept. of Psychology, University of California, Los Angeles, CA 90095, USA; g Brain Research Institute, University of California, Los Angeles, CA 90095, USA; h Department of Anatomy & Neurosciences, VU University Medical Center, Amsterdam, The Netherlands; i Brain Center Rudolf Magnus, Department of Psychiatry, UMC Utrecht, Utrecht 3584 CX, The Netherlands; j Department of Psychiatry, Massachusetts General Hospital, Boston 02114, USA; k Donders Institute for Brain, Cognition and Behaviour, Radboud University Medical Center, Nijmegen 6500 HB, The Netherlands; l Department of Psychology, Center for Brain Science, Harvard University, Cambridge, MA 02138, USA; m N.I. Vavilov Institute of General Genetics, Russian Academy of Sciences, Gubkin str. 3, Moscow 119991, Russia; n National Institute of Mental Health Intramural Research Program, Bethesda 20892, USA; o Neuroimaging & Cognitive Genomics Centre (NICOG), Clinical Neuroimaging Laboratory, NCBES Galway Neuroscience Centre, College of Medicine Nursing and Health Sciences, National University of Ireland Galway, H91 TK33 Galway, Ireland; p Institute on Aging, University of Florida, Gainesville, FL 32611, USA; q Department of Psychological Medicine and Psychiatry, Section of Addiction, King's College London, University of London, UK; r Departments of Neurosciences, Radiology, Psychiatry, and Cognitive Science, University of California, San Diego, La Jolla, CA 92093-0841, USA; s Departments of Neurosciences, Radiology, Psychiatry, and Cognitive Science, University of California, San Diego 92093, CA, USA; t Centre for Cognitive Ageing and Cognitive Epidemiology, Psychology, University of Edinburgh, Edinburgh EH8 9JZ, UK; u MRC-SGDP Centre, Institute of Psychiatry, King's College London, London SE5 8AF, UK; v Institute of Psychiatry, Psychology and Neuroscience, King's College London, UK; w Clinical Neuroscience Studies (CNS) Center, Department of Psychiatry, Icahn School of Medicine at Mount Sinai, USA; x Neuroimaging and Cognitive Genomics center (NICOG), School of Psychology, National University of Ireland, Galway, Ireland; y Language and Genetics Department, Max Planck Institute for Psycholinguistics, Nijmegen 6525 XD, The Netherlands; z Department of Psychiatry and Mental Health, University of Cape Town, Cape Town, South Africa; aa Department of Human Genetics, Radboud University Medical Center, Nijmegen 6525, The Netherlands; ab Department of Psychiatry, Radboud University Medical Center, Nijmegen 6525, The Netherlands; ac Department of Statistics, The University of Warwick, Coventry, UK; ad Psychiatry Department, University of Vermont, VT, USA; ae Department of Psychiatry, Yale University, New Haven, CT 06511, USA; af Olin Neuropsychiatric Research Center, Hartford, CT 06114, USA; ag Department of Psychiatry, University Medicine Greifswald, Greifswald 17489, Germany; ah Department of Psychiatry and Psychotherapy, HELIOS Hospital, Stralsund 18435, Germany; ai International Max Planck Research School for Language Sciences, Nijmegen 6525 XD, The Netherlands; aj Molecular Research Center for Children's Mental Development, United Graduate School of Child Development, Osaka University, Japan; ak Department of Radiology University Medicine Greifswald, Greifswald 17475, Germany; al Department of Psychiatry, University of Maryland School of Medicine, Baltimore, MD 21201, USA; am Department of Psychiatry, University of California, San Diego, La Jolla, CA 92093, USA; an Center for Human Genetic Research, Massachusetts General Hospital, USA; ao Department of Psychiatry, Harvard Medical School, USA; ap Stanley Center for Psychiatric Research, Broad Institute of MIT and Harvard, USA; aq Department of Psychiatry, University of Vermont, Burlington 05401, VT, USA; ar Groupe d'imagerie Neurofonctionnelle, UMR5296 CNRS CEA Université de Bordeaux, France; as QIMR Berghofer Medical Research Institute, Brisbane 4006, Australia; at Duke Institute for Brain Sciences, Duke University, NC 27710, USA; au Department of Statistics & WMG, University of Warwick, Coventry CV4 7AL, UK; av FMRIB Centre, University of Oxford, Oxford OX3 9DU, UK; aw Rotman Research Institute, Baycrest, Toronto, ON, Canada; ax Departments of Psychology and Psychiatry, University of Toronto, Toronto, Canada; ay Child Mind Institute, NY, USA; az The Hospital for Sick Children, University of Toronto, Toronto, Canada; ba Departments of Physiology and Nutritional Sciences, University of Toronto, Toronto, Canada; bb Center for Neuroimaging, Dept. of Radiology and Imaging Sciences, Indiana University School of Medicine, 355 W. 16th Street, Suite 4100, Indianapolis, IN 46202, USA; bc Center for Computational Biology and Bioinformatics, Indiana University School of Medicine, 355 W. 16th Street, Suite 4100, Indianapolis, IN 46202, USA; bd Department of Clinical and Experimental Epilepsy, UCL Institute of Neurology, London WC1N 3BG, UK and Epilepsy Society, Bucks, UK; be Department of Biological Psychology, VU University Amsterdam, Amsterdam, The Netherlands; bf Psychiatric and Neurodevelopmental Genetics Unit, Center for Human Genetic Research, Massachusetts General Hospital, USA; bg MRC Research Unit on Anxiety & Stress Disorders, South Africa; bh Neurogenetics Program, Department of Neurology, UCLA School of Medicine, Los Angeles 90095, USA; bi Institut Pasteur, Paris, 75015, France; bj Departments of Psychology and Neuroscience, Georgia State University, Atlanta, GA 30302, USA; bk Department of Psychiatry, VU University Medical Center (VUMC), Amsterdam, The Netherlands; bl Neuroscience Campus Amsterdam, VU/VUMC, Amsterdam, The Netherlands; bm Department of Psychiatry and Human Behavior, University of California, Irvine, CA 92617, USA; bn Department of Psychiatry and Psychotherapy, Charité Universitätsmedizin Berlin, CCM, Berlin 10117, Germany; bo School of Computing, Informatics and Decision Systems Engineering, Arizona State University, AZ 85281, USA; bp Brain Research Imaging Centre, University of Edinburgh, Edinburgh EH4 2XU, UK; bq Centre for Clinical Brain Sciences, University of Edinburgh, Edinburgh EH4 2XU, UK; br Queensland Brain Institute, University of Queensland, Brisbane 4072, Australia; bs Department of Computational Medicine and Bioinformatics, University of Michigan, Ann Arbor, MI 48109, USA; bt Department of Electrical Engineering and Computer Science, University of Michigan, Ann Arbor, MI 48109, USA

## Abstract

In this review, we discuss recent work by the ENIGMA Consortium (http://enigma.ini.usc.edu) – a global alliance of over 500 scientists spread across 200 institutions in 35 countries collectively analyzing brain imaging, clinical, and genetic data. Initially formed to detect genetic influences on brain measures, ENIGMA has grown to over 30 working groups studying 12 major brain diseases by pooling and comparing brain data. In some of the largest neuroimaging studies to date – of schizophrenia and major depression – ENIGMA has found replicable disease effects on the brain that are consistent worldwide, as well as factors that modulate disease effects. In partnership with other consortia including ADNI, CHARGE, IMAGEN and others^1^, ENIGMA's genomic screens – now numbering over 30,000 MRI scans – have revealed at least 8 genetic loci that affect brain volumes. Downstream of gene findings, ENIGMA has revealed how these individual variants – and genetic variants in general – may affect both the brain and risk for a range of diseases. The ENIGMA consortium is discovering factors that consistently affect brain structure and function that will serve as future predictors linking individual brain scans and genomic data. It is generating vast pools of normative data on brain measures – from tens of thousands of people – that may help detect deviations from normal development or aging in specific groups of subjects. We discuss challenges and opportunities in applying these predictors to individual subjects and new cohorts, as well as lessons we have learned in ENIGMA's efforts so far.

## Introduction

Here we provide an update on the progress of the ENIGMA consortium, a global alliance of over 500 scientists from over 200 institutions in 35 countries to study brain imaging data worldwide, discovering factors that modulate brain structure, integrity, connectivity, and patterns of brain differences in major brain diseases. Founded in 2009, ENIGMA's initial aims were to perform genome-wide analyses to identify common variants in the genome that are reliably associated with normal variability in brain structure. Since the initial effort discovered consistent effects worldwide of genetic variants that explained less than 1% of the variance in brain measures (Stein et al., 2015; Hibar and the CHARGE and ENIGMA2 Consortia, submitted for publication; Hibar et al., 2015a,b, in press), over 500 scientists have joined ENIGMA. ENIGMA is now (as of October 2015) a worldwide consortium, organized into over 30 working groups, studying major brain diseases (detailed at http://enigma.ini.usc.edu). The work in ENIGMA is divided into projects on (1) genetics, screening genomic data for predictors of individual variations in brain structure, function, and connectivity; (2) disease, screening brain measures to identify patterns of differences in the major brain diseases and factors that affect them; and (3) methods development. New “Big Data” methods are being developed and implemented around the world to perform genetic analysis of high-dimensional features that arise in neuroimaging — such as brain networks or “connectomes” (Sporns et al., 2005), 3D or 4D maps of brain changes over time, and more complex imaging data from functional MRI and EEG/MEG.

For this issue of NeuroImage we review the work ENIGMA has done, and how it relates to making individual predictions to support the emerging discipline of precision medicine — where personalized medical decisions are made considering an individual's genetic make-up, other risk factors, and the large body of scientific knowledge detailing genotype-phenotype relationships. ENIGMA's genetic and disease-related studies are discovering new factors that affect the brain throughout life, how the diseased brain differs from the healthy brain, and how patterns of brain measures differ from one disease to another. The potential to use machine learning methods in this context is vast, and we point to future opportunities and challenges, and what we have learned already about how individual genetic variants and diseases affect the brain.

One major thrust of ENIGMA's work is genomics, so we first review studies that discovered individual loci in the genome that are linked to variations in brain structure (Stein et al., 2012; Hibar and the CHARGE and ENIGMA2 Consortia, submitted for publication; Hibar et al., 2015a,b, in press). The effect of these common genetic variants tends to be small, but the aggregate effect of thousands of them accounts for a substantial proportion of the variance in brain measures (Toro et al., 2015; Ge et al., 2015; Chen et al., 2015). The relevant genes can be difficult to discover in individual cohorts, but they can be detected by meta-analyzing data across multiple sites. We discuss multivariate and machine learning methods needed to combine some of these predictors in more powerful models that can make valuable predictions about individuals, such as predicting deviations from normal lifetime aging, risk for mental illness, or recovery from trauma.

### Reproducibility

There have been numerous recent surprises regarding the nature of gene effects on the brain, including surprisingly poor reproducibility of candidate gene effects on imaging measures and risk for mental illness, and the very large sample sizes needed to reliably detect any genetic associations at all. There have also been dramatic claims of poor reproducibility of findings in genetics, neuroimaging, and neuroscience studies in general (Button et al., 2013; Ioannidis, 2014; Ioannidis et al., 2014). Meta-analyses, such as those conducted by ENIGMA, have been proposed as a way to screen for false positive findings. If claims of “significance chasing” and “fishing” in neuroscience studies are true (Ioannidis, 2014), then predictive models based on them should fail more often than models based on meta-analyzed studies of large numbers of independent cohorts, analyzed in a harmonized way (Ware and Munafò, 2015). ENIGMA is dedicated to replication, and a number of initiatives are underway to develop methods to replicate imaging genomics findings.

We discuss factors that affect reproducibility of models that predict specific gene effects on the brain, including technical factors of image acquisition and analysis. Low effect sizes for individual predictors make genetic effects hard to detect, so meta-analysis is valuable in demonstrating effects that no single cohort can detect on its own. Clearly, if we build a model to classify a person into a certain diagnostic group, based on a set of predictors, we also need to know how to decide if we have measured the predictors well enough, or if the context where the model was fitted is similar enough to the current situation for the prediction to make sense and be accurate. Apart from the choice of predictive model and predictors, there are many other reasons why imaging or genetic models of diagnosis or prognosis may generalize poorly or not at all, depending on the context. Factors that affect model prediction will include age and environment, and the demographic history of the populations sampled; these may affect whether or not a predictor is relevant to a new cohort or an individual. In the ENIGMA studies below, we point to examples in which predictors in the genome and image would be valuable in making individual predictions about brain volume or about a person's diagnosis, but only in certain contexts, such as in certain parts of the lifespan, or only after considering certain confounds or variables that are known to drive brain differences (duration of medication and duration of illness are often confounded, and modeling each effect independently may produce paradoxical conclusions, e.g., that medication is bad for the brain). Individual predictive models are likely to become increasingly nuanced, as we find out more about how predictors interact and contexts where different models work best.

In the course of ENIGMA's efforts, a vast quantity of normative data has been gathered and analyzed from different countries and continents of the world, allowing us to make some inferences about the normal trajectory of brain development and aging (ENIGMA-Lifespan; Dima et al., 2015). We discuss the challenges and opportunities in using models based on these data to make assertions about individual and group deviations from normal, or to generate cohort, or national norms, if they exist and if their value outweighs the costs of generating them.

We also discuss several concepts that have increased the power of ENIGMA to find factors with very small effects on the brain, including how we assess their generality and extensibility to new cohorts.

## ENIGMA's Genetic Studies

By December 2009, many researchers worldwide had collected genome-wide genotyping data from cohorts of subjects for whom brain imaging information such as anatomical MRI was available.

It had long been presumed that genetic and environmental factors, and the complex interactions among them, play a role in shaping brain structure. Decades of work in behavioral and medical genetics had convincingly shown that many of the major brain diseases – from Alzheimer's and Parkinson's disease to psychiatric illnesses such as schizophrenia and major depression – had a strong additive genetic component. Similar genetic risks exist for neurodevelopmental disorders such as autism. Even so, studies of identical twins who share the same genome show that genetic factors do not fully account for disease risk, and discordant twin pairs provide valuable information about the impact of environmental and epigenetic factors on disease (Munn et al., 2007). Furthermore, many common disorders are likely to reflect a constellation of modest gene differences acting in concert, which smaller individual studies are unlikely to find. Instead, larger studies that capture heterogeneity have begun to unravel the influence of multiple ‘low level’ minor but important gene differences on disease expression (Lopez et al., 2015).

**Fig. 1:**
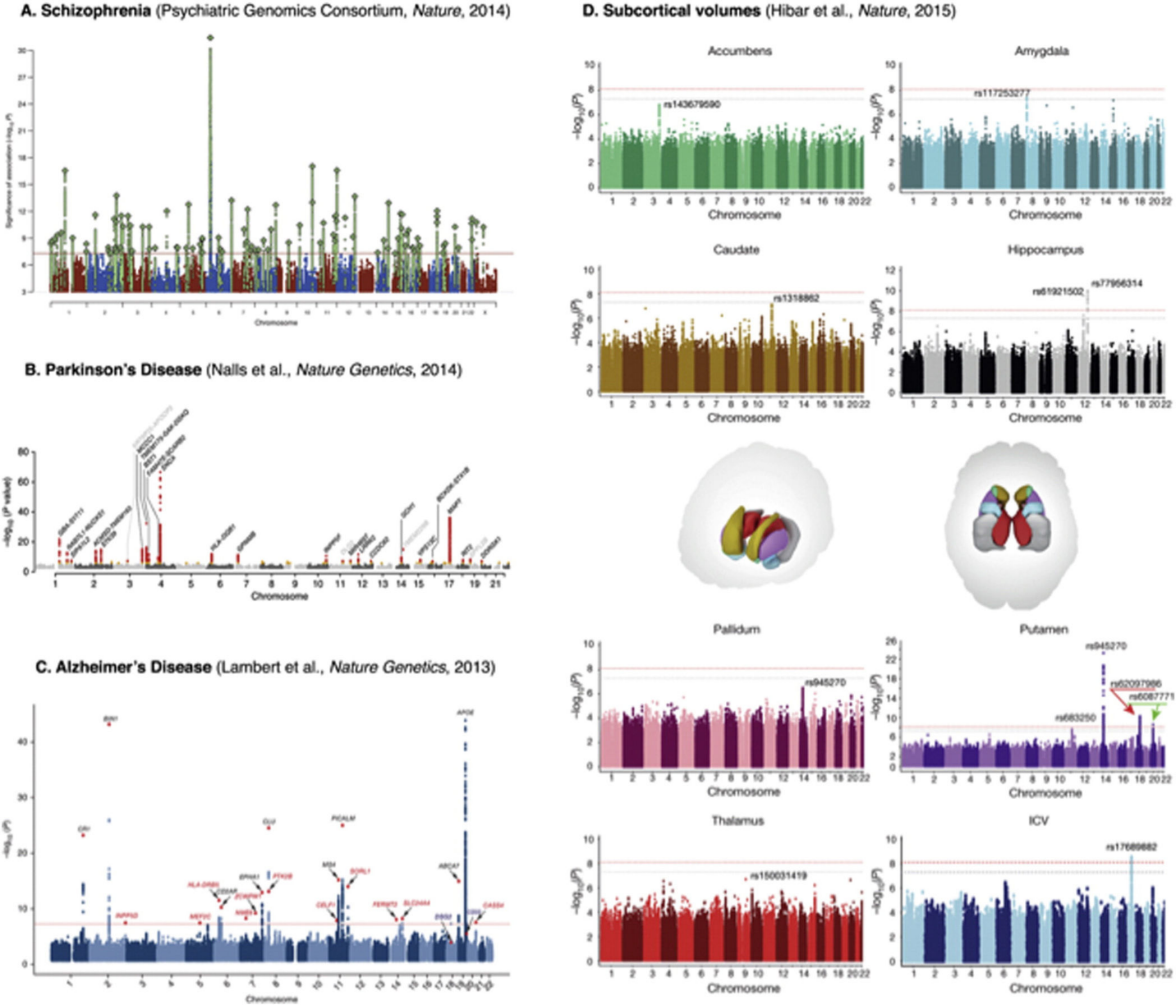
Recent genome-wide association studies (GWAS) of brain disorders and brain structure. **Part A** shows the Manhattan plot from a 2014 *Nature* meta-analysis conducted by the Psychiatric Genomics Consortium. The genetic variants are presented on the *x*-axis, and the height of the dots shows the strength of association between each genetic variant and schizophrenia. A negative log *p*-value scale is used: higher points denote stronger associations. The group identified 108 schizophrenia-associated genetic loci in a sample of 34,241 cases and 45,604 controls (*red line* = genome-wide significance level, conventionally set at p = 5×10^–8^; *green SNPs* = polymorphisms in linkage disequilibrium with index SNPs (diamonds), which indicate independent genome-wide significant signals). **Part B** 26 loci significantly associated with risk of Parkinson's Disease (Nalls et al., 2015), in 13,708 cases and 95,282 controls (*red SNPs* = genome-wide significant signals). **Part C** 19 loci significantly associated with risk of AD, in a sample of 17,008 cases and 37,154 controls (Lambert et al., *Nature Genetics*, 2013; genes identified by previous GWAS are shown in black; newly associated genes in red; red diamonds indicate SNPs with the smallest overall *p*-values in the analysis). **Part D** shows genome-wide associations for eight subcortical structures, conducted by the ENIGMA consortium in 30,717 individuals from 50 cohorts worldwide (Hibar et al., *Nature*, 2015). This study identified five novel genetic variants associated with differences in the volumes of the putamen and caudate nucleus and stronger evidence for three previously established influences on hippocampal volume (see Stein et al., *Nature Genetics*, 2012) and intracranial volume (see Ikram et al., *Nature Genetics*, 2012). Each Manhattan plot in Part D is color-coded to match its corresponding subcortical structure, shown in the middle row. The gray dotted line represents genome-wide significance at the standard p = 5×10^–8^; the red dotted line shows a multiple-comparison corrected threshold of p = 7.1 × 10^–9^. [Images are reproduced here with permission from MacMillan Publishers Ltd (*Nature Genetics*, 2012 & 2013; *Nature*, 2014 & 2015) and with permission from the corresponding authors.]

As high-throughput genotyping methods became available, *genome-wide association studies* (GWASs) began to reveal specific sources of risk in the genome for several major brain diseases (Fig. 1). To fully appreciate this kind of study, we need to understand that much of the genome is invariant between humans (Rosenberg et al., 2002). Many kinds of individual genetic variations – common or rare – can occur, including polymorphisms, insertions and deletions of genetic material, loss or retention of homozygosity (LOH/ROH), or copy number variations (CNVs) — where the number of copies of pieces of genomic material differs from the normal two alleles in some individuals but not others. Polymorphisms are a common marker of individual differences, where a single nucleotide polymorphism (SNP) is essentially a “single-letter” change in the genome: a change in a single base pair between individuals.

Some genomic changes interfere with the viability of the organism, leading to very low frequencies in the population. Others remain and some have a moderate or severe impact on a person's health, or their risk for disease. For example, a common variant (present in 1 in 100 in the general population) in the *HFE* gene impairs a person's ability to metabolize iron. Excessive iron levels can then accumulate in bodily organs, which can cause liver and kidney failure. Multiple deletions in the 22q region of the genome provide another example. Individuals with these deletions have a characteristic neurodevelopmental profile associated with mild to severe abnormalities in the face, brain, and heart, and are at heightened risk for schizophrenia and autism. 22q deletions occur frequently *de novo*, so they do not really remain in the population; rather 22q is a vulnerable spot in the genome for mutation. Even so, 22q deletion syndrome – and other neurogenetic disorders such as Fragile X, Williams syndrome, and Turner syndrome – have often been studied to help identify potential mechanisms that may contribute to more prevalent psychiatric conditions. ENIGMA's 22q working group has been set up to understand brain differences associated with deletions at this locus, and how they relate to those found using the same analysis protocols in ENIGMA-Schizophrenia and ENIGMA-Autism.

Genetic risk for many major psychiatric illnesses is thought to be mediated in part by common genetic variants that have persisted in human populations for thousands of years. In many cases, the adverse effects of disease risk genes – such as the Alzheimer's risk gene, *APOE* – are not apparent until later in life (Hibar and the CHARGE and ENIGMA2 Consortia, submitted for publication; Hibar et al., 2015a,b, in press). Because of this, the variants tend to be preserved in the gene pool and continue to drive disease risk worldwide.

Geneticists continue to debate the relative contribution of common versus rare genetic variants to risk for various diseases, but a recent large-scale screen of schizophrenia patient cohorts worldwide implicated over 100 genetic loci in risk for the disease (Ripke et al., 2014; Fig. 1). This highly successful study pointed to several genes in the dopamine neurotransmission pathway that had long been implicated in schizophrenia and its treatment — for example, a functional polymorphism in the *DRD2* promoter region, which modulates levels of gene expression, and affects antipsychotic drug efficacy (Zhang and Malhotra, 2013). This same genomic screen pointed to other unexpected genetic variants in immune system pathways that offer tantalizing new leads about disease mechanisms, and the role of modifiable factors in eventually treating or averting the illness. Similar efforts in bipolar illness, major depression, and ADHD uncovered genes driving risk for these disorders that overlapped to some extent with those for schizophrenia and with each other (Cross Disorders Working Group of the Psychiatric Genomics Consortium, 2013). Members of the ENIGMA Consortium have recently demonstrated the usefulness of polygenic risk scores for schizophrenia (based on the 108 loci shown in Fig. 1A) in revealing an association between early cannabis use and brain maturation during adolescence — replicated in three samples (French et al., 2015).

Many successful genomic screens involve over 100,000 individuals. For example, the most recent GWAS of height, educational attainment, and body mass index (BMI) identified 56 novel BMI-associated loci in a sample of up to 339,224 individuals (Wood et al., 2014; Locke et al., 2015). Similarly, the Psychiatric Genomics Consortium's discovery of genetic loci implicated in schizophrenia risk took a ‘quantum leap’ once the sample sizes exceeded 75,000 (Ripke et al., 2014), after less successful searches in smaller samples. Several factors may contribute towards this need for large sample sizes in genome-wide association. First, there are biological variation and ascertainment differences among cohorts. A person diagnosed with a specific illness may have other co-morbid illnesses, and diagnostic criteria may vary somewhat worldwide in terms of who is included in the groups of patients and controls.

However, the main reason GWAS needs large samples is power: a genome-wide association analysis comprises approximately a million independent tests, so a threshold of *p* < 5 × 10^–8^ is employed to minimize false positives. Early GWAS estimated their required sample sizes based on published effect sizes of candidate genes that have since been shown to be greatly overestimated. Although the genetic architecture of each trait is unique, for most complex traits the effect sizes of individual SNPs are typically less than half a percent (Franke et al., in press). Thus, it follows from power analyses that GWAS and GWAS meta-analyses typically require data from tens of thousands of individuals.

In the imaging field, initial studies also attempted genome-wide screens of brain imaging measures, such as brain size (Paus et al., 2012), the volume of the temporal lobes on MRI (Stein et al., 2010a,b), in cohorts of around 800 subjects (see Medland et al., 2014, for a review). This type of analysis became feasible as large cohort studies, such as the Alzheimer's Disease Neuroimaging Initiative (Jack et al., 2015), started to put their images and genomic data online. In line with accepted practice in genetics, it is customary to require replication of such genetic effects in independent cohorts.

While some effects appeared to replicate, most did not as the studies were underpowered, and it was unclear whether cohort factors, biological differences, or technical factors were to blame.

### Endophenotype Theory and Power

As the field of imaging genetics grew, some researchers hoped that imaging might offer a more efficient approach to discover genes involved in mental illness. The reason for this optimism was based on the observation that many brain measures are consistently reported as affected in psychiatric cohort studies (see later, under *ENIGMA Disease Studies*), so they could maybe serve as quantitative traits, or markers, correlated with the illness.

There was also some hope that the biological signals in images – measures of neurotransmitters, receptors or metabolite levels, blood flow, the volume of specialized brain areas such as the hippocampus, or its chemical content – might be influenced by genetic variants because of their proximity to primary gene action. Likewise, it was argued that brain-derived measures may have a simpler genetic architecture – perhaps with fewer individual genes or pathways influencing them – compared to the multitude of factors driving a person's overall risk for developing a disease (Saykin et al., 2015). Brain measures may also offer a more precise or reproducible diagnostic scale. Potkin et al. (2009) noted that GWAS can be more efficient when researchers analyze continuous measures (such as brain volumes) rather than binary traits, such as diagnosis, which may also disguise complexities such as co-morbidity, etc.

This endophenotype theory^2^ led to confidence that genome-wide screening of brain measures would yield “hits” – genetic loci consistently associated with brain measures – relatively efficiently and, some believed, in much smaller samples. Several countervailing arguments should also be considered. The genetics of brain traits may reveal common pathways involved in a number of mental illnesses, but one loses some specificity when moving from a psychiatric disorder to brain measures — different disorders may have very similar brain abnormalities. For this reason, ENIGMA's Disease Working groups have analyzed tens of thousands of brain scans to see which measures best distinguish patients from controls, across a range of 12 diseases, with a view to understanding similarities and differences. Collecting brain imaging data is more expensive than diagnostic testing. Also, genes that affect brain measures may be of less interest to a patient or physician unless they are also connected to disease risk or prognosis. In ENIGMA, however, the costs of collecting the imaging data had already been incurred, making the feasibility of a large-scale analysis the main consideration. Others voiced a muted optimism: Munafò and Flint (2014) noted that effect sizes for gene effects on neuroimaging data were not likely to be any greater than for any other trait, but the value in studying them came from the ability of brain measures to help understand mechanisms that might underlie associations between genes and more conventional traits (see also Flint et al., 2014). Yet, the potential to find genetic factors that jointly influence risk for mental illness and a neuroimaging trait could dramatically improve statistical power and provide an important link between the genome and the behavioral symptoms used to diagnose psychiatric and neurological illnesses (Glahn et al., 2014).

In ENIGMA's first paper in *Nature Genetics*, Stein and 158 authors (2012), including 4 existing consortia (SYS, EPIGEN, ADNI, and IMAGEN^3^), meta-analyzed GWAS data from cohorts worldwide and found genetic loci consistently associated with the size of the human hippocampus and total intracranial volume. Notably, in a partnership with another consortium, CHARGE (Bis et al., 2012), the top “hits” – the genetic variants with greatest effect sizes – were anonymously exchanged and found to be the same, supporting the replicability of the findings in completely independently designed efforts.

In a follow-up study in a larger sample (*N* = 21,151 individuals; Hibar and the CHARGE and ENIGMA2 Consortia, submitted for publication; called “ENIGMA2”), eight genetic loci were discovered that were reliably associated with the size (volume) of several subcortical structures, including the putamen, caudate, and pallidum. With the increased sample size, earlier findings regarding the hippocampus and intracranial volume were replicated and reinforced; new genetic loci were also discovered. Several of the SNPs implicated lie within or close to genes involved in cell migration, axon guidance, or apoptosis — all cellular processes likely to lead to observable differences in the size of cellular nuclei in the brain. Parallel work in mice by the Williams lab in Memphis began to study mouse homologs of these variants (Ashbrook et al., 2014); recent data suggest that variation of the top putamen gene, *KTN1*, can predict putamen volume and cell counts in outbred mice (R. Williams, *pers. commun.*).

Several lessons were learned from the first two ENIGMA genetic studies, in addition to a third pair of papers currently in submission, involving an even larger sample (*N* > 31,000; Hibar and the CHARGE and ENIGMA2 Consortia, submitted for publication; Hibar et al., 2015a,b, in press; Adams and the CHARGE and ENIGMA2 Consortia, submitted for publication). First, through meta-analyses, it was possible to detect factors (here, SNPs) that accounted for less than 1% of the variance in brain measures. This was despite the fact that the participating studies were designed with different goals in mind, and many used scanners of different field strengths, processed by researchers who had not all met, and communicated through email and teleconference calls.

Much of the consistency in brain measures capitalized on the ongoing refinement of standardized protocols for analyzing images and genomes; in turn, those protocols relied on decades of work by developers of widely used and extensively tested analysis packages such as FreeSurfer (Dale and Sereno, 1993; Fischl, 2012), and FSL (Jenkinson et al., 2012). The supplement of the first ENIGMA paper (Stein et al., 2012) contained 104 pages of ancillary tests supporting the validity and reliability of the data, including tests comparing different imaging software for brain volume quantification.

On the genomic side, the ability to compare genomic data in a common reference frame depended on the availability of the HapMap3 (The International HapMap3 Consortium, 2010) and later the 1000 Genomes reference datasets (Genomes Project C et al., 2010). These reference panels are continually updated and refined, and allow genotyping data collected with one kind of genotyping array (“chip”) to be imputed to match data collected using others, and pooled in the same overall study.

A second issue is whether these findings could have been detected more efficiently using only some of the samples. In a sense, this is a “meta-question” — how might the study have been designed more efficiently after seeing the results?

As in any meta-analysis, the weight assigned to each cohort in the final statistics can be made to depend on its total sample size, or on the standard error of the regression coefficients (which is in fact what ENIGMA does). As such, it is not vital for every cohort to reject the null hypothesis on its own. In fact, any cohort study, however small, can partner with other sites to contribute to the discovery of effects that it cannot detect alone. In ENIGMA1 (Stein et al., 2012), only 5 of the 21 cohort studies were able to detect the effect of the SNPs on the brain in their cohort alone, at the nominal significance level of *p* = 0.05. By the time of ENIGMA2, 20 of the 38 Caucasian European (CEU) cohort studies could detect the effects of the top SNP. Even so, the aggregate support of the discovery and replication samples was crucial to making sure the effects were credible and unlikely to be false positives.

### Relevance to Disease Risk

The quest to identify genetic variants associated with brain measures is partly motivated by finding variants that affect our individual risk for disease. Any modulators of health outcomes in populations may have a vast impact on society, even if they are not the main factors explaining risk for any one individual. As well as affecting risk for disease, genetic differences may also affect symptom severity, treatment response, and prognosis.

As such, several clinical trials for Alzheimer's disease drugs already stratify their cohorts by *APOE* genotype — a major risk gene for AD that may have a bearing on treatment response as well as disease risk (see Riedel et al., submitted for publication, for a review of *APOE* effects, which are remarkably complex). At the time of writing, several manuscripts are under review addressing the overlap between ENIGMA's genomic findings and accepted or emerging markers of disease risk (Hibar and the CHARGE and ENIGMA2 Consortia, submitted for publication; Hibar et al., 2015a,b, in press; Adams and the CHARGE and ENIGMA2 Consortia, submitted for publication; Franke et al., in press). Here we simply review their overall design. Some initial reports have appeared in abstract form, relating brain-related SNPs to risk for Parkinson's disease (Hibar and the CHARGE and ENIGMA2 Consortia, submitted for publication; Hibar et al., 2015a,b, in press), obsessive compulsive disorder (Hibar and the CHARGE and ENIGMA2 Consortia, submitted for publication; Hibar et al., 2015a,b, in press), schizophrenia (Stein et al., 2015; Franke et al., in press), and multiple sclerosis (Rinker et al., submitted for publication). An initial negative report has appeared for epilepsy (Whelan et al., 2015). Even so, given the low fraction of heritability explained by the SNPs discovered, the studies so far are widely accepted as underpowered.

One method to assess an individual's relative risk for disease, based on genome-wide genotyping data, involves computing a polygenic risk score (PRS) for each individual. In Alzheimer's disease, for example, carrying one copy of the *APOE4* genotype boosts lifetime risk for AD by a factor of 3, and carrying two copies may boost risk by 15 times. These odds ratios are not constant across human populations and even vary by ethnicity, or circumstances, so some caution is needed when extrapolating them to new data; but as AD GWAS data accumulate, over 20 common genetic variants have been found to affect AD risk — 3 of them, in the genes *CLU, PICALM*, and *CR1*, appear to be associated with a difference in disease risk of over 10% per allele. If an individual's genotype is known for these loci, it is possible to create a polygenic risk score in a number of different ways, depending on whether the goal is to predict diagnosis, outcome, or brain measures. The simplest approach is to count risk loci, although that clearly ignores the vastly different odds ratios from each locus. It is more common to weight the loci based on their odds ratio for disease, or by their regression coefficients. *APOE4*, for example, is just a single genotype that might contribute to calculation of a polygenic risk score together with other risk loci. As shown by the PGC analyses, the predictive accuracy of PRS scores increases as the number of variants included increases. Calculation of these scores does not need to be restricted to genome-wide significant loci.

Recent efforts to predict disease status based on polygenic risk scores have had varied success, but the reasons are quite well understood. First, for the most prevalent neurological or psychiatric diseases, we do not yet have a set of common variants that account for more than a small fraction of disease risk (except for *APOE4*, where a single copy may triple a person's risk for AD, other factors being equal). In AD, there are rare mutations in genes related to AD pathology – such as presenilin and APP – that invariably produce early-onset AD. Carriers of these genetic variants are the targets of major neuroimaging initiatives (Benzinger et al., 2013). A very important aspect of this – relevant to the field of *personalized medicine* – is that the person's genotype in conjunction with amyloid imaging can accurately predict the age of onset for the disease and the symptoms (Benzinger et al., 2013).

Another cause for optimism is the efforts of the Psychiatric Genomics Consortium (PGC). When the PGC Schizophrenia Working Group increased their sample size to 36,989 cases and 113,075 controls, they discovered over 100 loci associated with risk for schizophrenia, suggesting that other GWAS may experience similar boosts, depending on where they are in the arc of discovery. The rate of success of these efforts, and yield on the efforts invested, also depends on the polygenicity of each disease, and the distribution of risk loci across the genome. Holland et al. (submitted for publication) used recent data from the ENIGMA study and the PGC to estimate what sample sizes are needed for a GWAS to discover enough SNPs to account for, say 50% or 80% of the chip-based heritability, i.e., the amount of the population variance predictable from genotyped SNPs. They argued that some traits are more polygenic than others, and that, relative to some brain measures, GWAS studies of schizophrenia and major depressive disorder may require much larger sample sizes to discover enough SNPs to account for high levels of the chip-based heritability. If that is true, then imaging genetics may be well on the way to a significantly higher rate of discovery, and a more complete understanding of common variants driving individual differences in brain measures.

### How much individual variance is explainable by GWAS and common genetic variants?

In recent years, a number of powerful methods emerged to estimate what fraction of the population variance in a trait could be predicted, in principle, from all the SNPs on the genotyping chip, even if the exact genes and SNPs were not yet known.^4^ Predictions can be made from the full set of association statistics: models (linear or Gaussian) are first fitted to the observed effect sizes of *all* the SNPs, even if most SNP effects fail to reach the accepted standard for genome-wide significance. In much the same way as FDR (the false discovery rate method) is used in imaging to confirm evidence for a distributed signal — spread out across the brain, the overall effect of genome-wide SNPs on a trait can be estimated without having to pinpoint which exact regions — of the image or the genome — contribute unequivocally to the effect.

Hibar et al. (2015) used genome-wide summary statistics to estimate heritability (So et al., 2011) and found that common variants across the genome explained around 19% of the variance in hippocampal volume, which is comparable to SNP-based estimates of heritability for many psychiatric disorders and other biological traits. More recently, B.K. Bulik-Sullivan et al., 2015 introduced a similar method based on linkage disequilibrium^5^ (LD) scores that is also able to recover heritability from summary statistics. The LD score method assigns an LD score to each SNP — the sum of its squared correlations (*r*^2^) with all other SNPs in a 1 centimorgan window. One then regresses the chi-squared statistics from a GWAS against the LD score for each SNP. The slope of the resulting regression line depends on the sample size and the SNP-heritability — the proportion of trait variance accounted for by all the genotyped SNPs (see B. Bulik-Sullivan et al. (2015), B.K. Bulik-Sullivan et al., 2015, for derivations).

A related method, GCTA (genome-wide complex trait analysis; Yang et al., 2011) suggested that a still higher proportion of population variance in brain volumetric measures may be accounted for based on all genotyped SNPs, even in cases where we do not know which SNPs help as predictors of the trait. Members of the ENIGMA Consortium have applied this method to estimate SNP-based heritability for structural (Toro et al., 2015) and functional (Dickie et al., 2014) brain measures. A working group in ENIGMA, ENIGMA-GCTA, is now comparing the GCTA and LD score methods to better estimate how much brain variation is explainable by genotyped SNPs, at least for the brain measures that are most readily computed from MRI. SNP-based heritability estimates of cortical surface area for different cortical subdivisions calculated by GCTA were recently published (Chen et al., 2015). These cortical subdivisions were defined by a genetically based cortical parcellation scheme (Chen et al., 2012).

The reason ENIGMA and other GWAS researchers are interested in measuring heritability – and ideally the fraction of heritability explained by common genetic variants – is that it should be possible to prioritize brain measures for deeper genetic analysis based on their heritability, reliability, polygenicity, and relevance to disease. Such rankings or “Bayesian priors” would help in prioritizing research, making studies more efficient and better powered (Schork et al., 2013; Becker et al., submitted for publication; Holland et al., submitted for publication; Wang et al., submitted for publication). Even so, there is no evidence that phenotypes with higher heritability show stronger associations with SNPs. One such example is white matter hyperintensities — a brain measure with high heritability, for which specific genomic risk factors have been hard to find. The main benefit of focusing on highly heritable phenotypes comes from the fact that measurement error is typically lower, and prioritizing brain measures is important as there are so many ways to quantify brain structure and function.

A recurring caveat in this work is that the SNP effects are not expected to be constant in all cohorts. They may depend on a person's age, environment, or other circumstances. We now know from ENIGMA2 that the top 8 loci associated with the volumes of subcortical structures were detectable consistently worldwide, even though each one accounts for < 1% of the variance. A later screen for age × SNP effects suggested that some genes have a greater effect on brain measures later in life (Hibar and the CHARGE and ENIGMA2 Consortia, submitted for publication; Hibar et al., 2015a,b, in press), perhaps because they interact adversely with other biological processes or environmental stressors. In other words, although ENIGMA primarily uses meta-analysis to assess evidence, we do not assume that the effect size is always the same. Heterogeneity of effects is also assessed – a SNP effect important late in life may not be replicated in younger samples. Conversely, since most psychiatric disorders occur at a young age, one may expect to find associations that link genetic vulnerability, brain structure and disease at a younger age, with effects that may diminish later. Moreover, for certain disorders such as addiction, the psychological, neurobiological and genetic factors most relevant at one age (e.g., impulsivity or sensation-seeking in adolescents experimenting with drugs) may be quite different from the factors when dependent (e.g., compulsivity or habit-based behavior) or when recovering (e.g., stress regulation or cognitive control). Even so, ENIGMA's genomic screens so far are only well-powered to detect SNP effects that are consistent — there may also be SNP effects, so far undetected, that depend on the demographics of the cohort assessed, or disease status, or other circumstantial factors.

This is a reminder that predictive models work best in cohorts similar to those where discoveries were made. Because of this concern, which to some extent affects all brain imaging studies — and all human studies — ENIGMA has diversified to over 33 countries. Recently, ENIGMA partnered with other consortia such as the Japanese consortium, COCORO (Okada et al., in press); encouragingly, effects of psychiatric illness on brain structural measures were replicated in Western and Eastern populations, not just in the structures affected the most, but in their rank order, showing congruence between independent studies (van Erp et al., 2015; Okada et al., in press).

## ENIGMA's Disease Studies

After the initial success of the genetic analyses (Stein et al., 2015; Hibar and the CHARGE and ENIGMA2 Consortia, submitted for publication; Hibar et al., 2015a,b, in press), ENIGMA investigators had analyzed brain MRI data from well over 30,000 individuals — around a third of the data came from patients with a range of psychiatric conditions. In the primary GWAS studies, analyses were run with and without patients, and excluding patients did not affect the main findings; of course the possibility remains that some SNP effects may be easier to detect in some patient cohorts, but ENIGMA's overall results were not driven by the presence of patients.

**Fig. 2:**
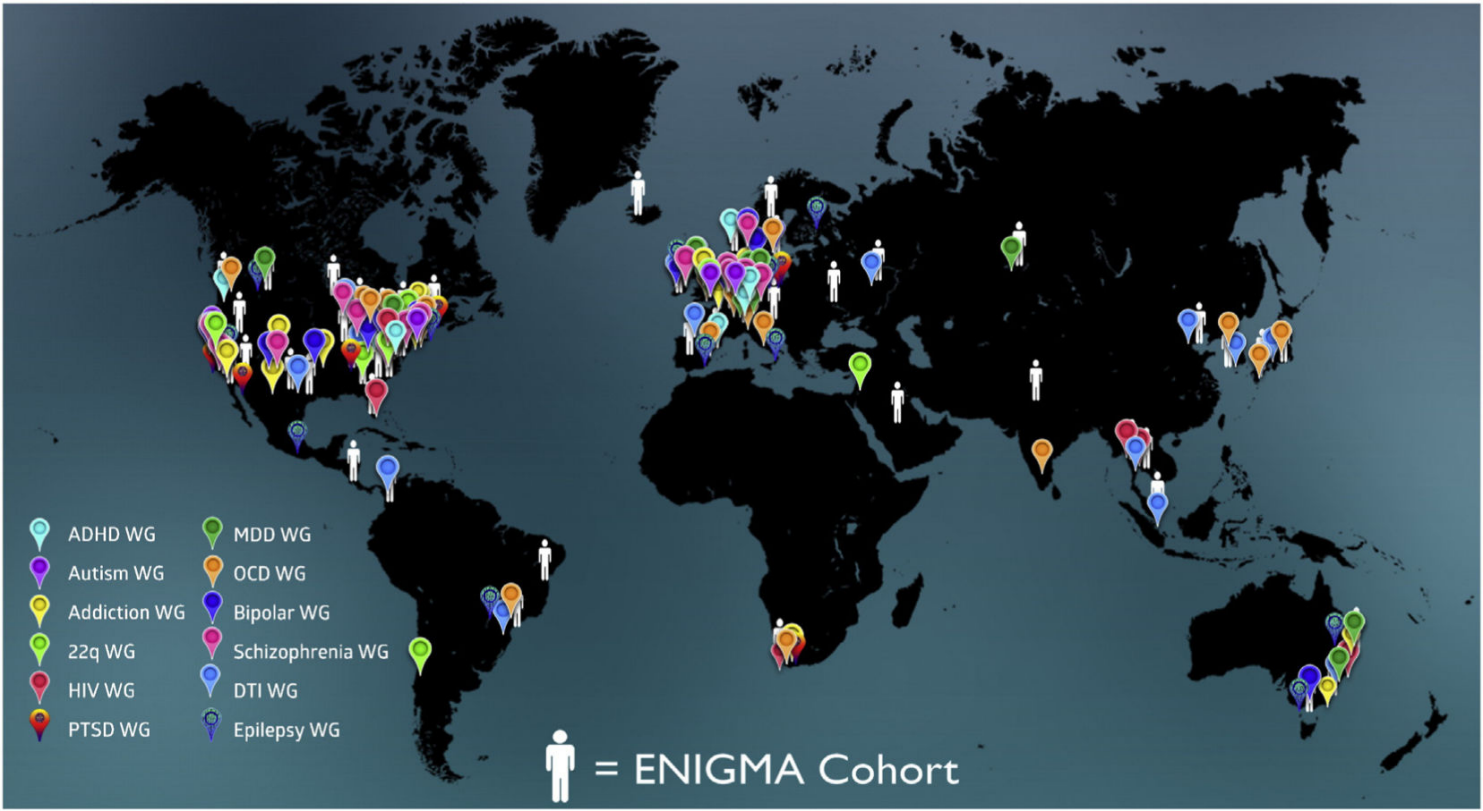
ENIGMA Map The ENIGMA consortium now consists of over 30 Working Groups made up of 500 scientists from over 200 institutions and 35 countries; several of these Working Groups have several ongoing secondary projects, led by different investigators. Here we show 12 of the working groups, focusing on specific diseases and methodologies, including ADHD, autism, addiction, bipolar disorder, diffusion tensor imaging, epilepsy, HIV, major depressive disorder, OCD, PTSD and schizophrenia. Centers where individuals are scanned and genotyped are denoted with color-coded pins (*legend, bottom left*).

**Fig. 3:**
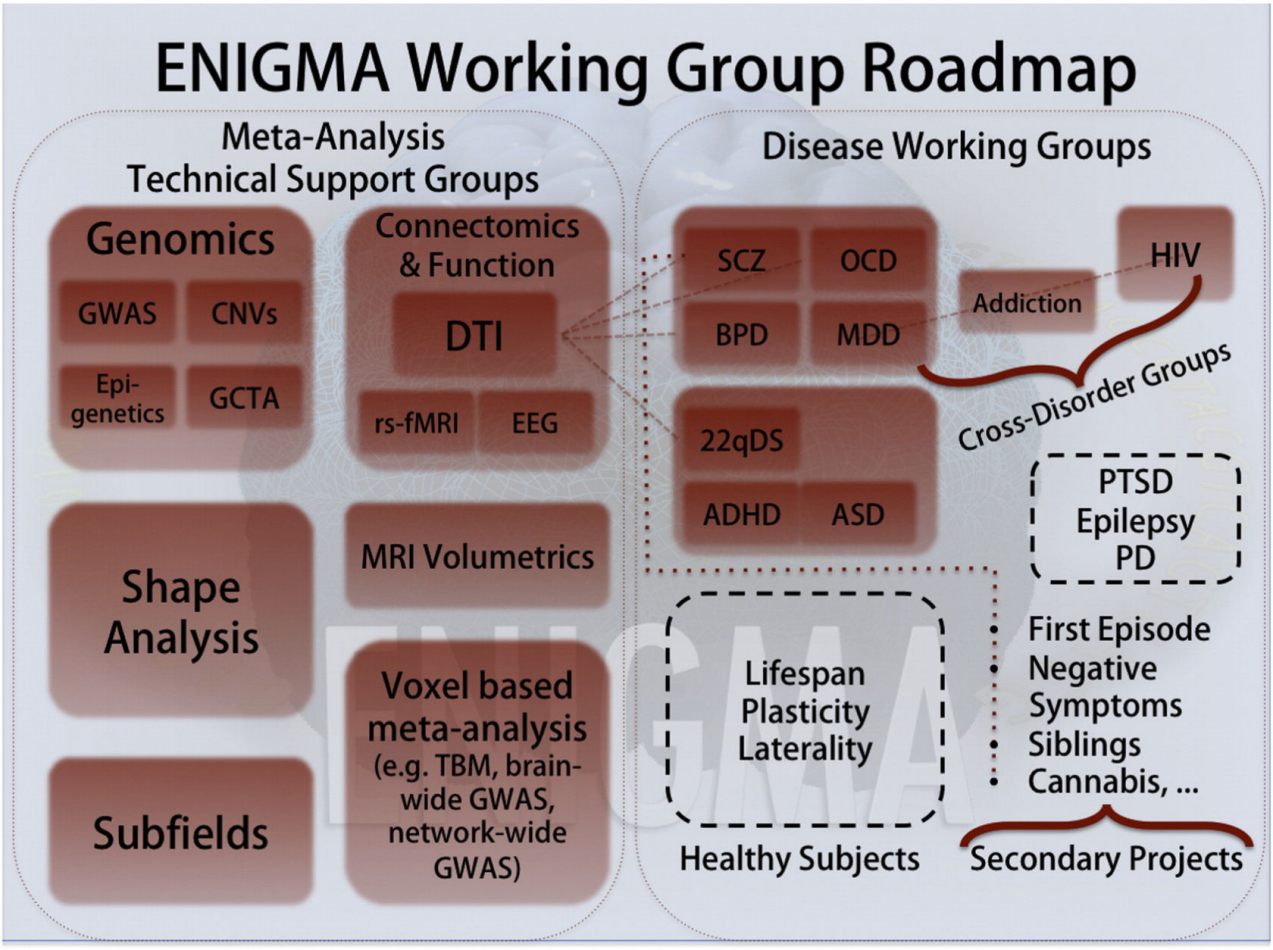
ENIGMA Roadmap The current organization of ENIGMA's Working Groups is shown here. Several groups relate brain measures to variation in the genome, and specialized groups are dedicated to helping members run analyses of genome-wide SNP data, copy number variants, and epigenetic markers on the genome. In parallel, there are psychiatric and neurology working groups dedicated to the study of worldwide data from a range of diseases. As shown here in detail for the schizophrenia working group, there are secondary projects, to relate brain variation to specific symptoms or clinical measures. In parallel, support groups coordinate large scale efforts to harmonize DTI (diffusion tensor imaging) and related brain data (Jahanshad et al., 2014). Partnerships between the DTI and Genomics groups are leading to genome-wide screens of DTI measures in over 13,000 people; cross-disorder partnerships study brain features that may relate to diagnostic boundaries, or common co-morbidities, allowing factors driving brain variations to be disentangled.

In 2012, ENIGMA formed working groups on schizophrenia (van Erp et al., 2015), bipolar disorder (Hibar and the CHARGE and ENIGMA2 Consortia, submitted for publication; Hibar et al., 2015a,b, in press), major depression (Schmaal et al., 2015), and ADHD (Hoogman et al., 2015); groups meta-analyzing data on 8 additional disorders have been formed since, with current sample sizes detailed in Table 1; a map of participating sites is shown in Fig. 2. In the summer of 2015, additional working groups were formed on anorexia nervosa, recovery after stroke, and Parkinson's disease — the current “roadmap” showing relationships between ENIGMA's working groups is shown in Fig. 3 (also see http://enigma.ini.usc.edu for the latest status). The diseases surveyed include many where controversy exists on the nature and scope of disease effects on the brain. Given this controversy, the main benefit of meta-analysis is to discover which effects are strongest or most reliably found, and which depend on known or unknown factors of the cohorts assessed.

The initial goal of ENIGMA's Disease working groups has been to meta-analyze effects of these disorders on the subcortical brain measures studied in the GWAS study. As scans had already been analyzed with a harmonized protocol, and subtle genomic effects had been discovered, there was some interest in ranking brain measures in terms of disease effects (i.e., differences between patients and controls).

A secondary goal was to find factors that might moderate how these diseases impact the brain, such as a person's age, the duration or severity of illness, comorbidities, or treatment-related effects, such as which medications the patients had been treated with, and for how long. Clearly, treatment effects on the disease or the brain depend on many factors. ENIGMA's multiple cohorts, in some cases, offered the opportunity to gauge their generality or consistency. At the same time, many groups joined ENIGMA and provided only brain measures as their initial case–control analyses did not require genome-wide genotyping data on their cohorts. As such, truly vast samples began to be analyzed (*N* = 8,927, in the published ENIGMA-Depression study; *N* = 10,194 in the ENIGMA-Lifespan study; see Table 1).

**Fig. 4:**
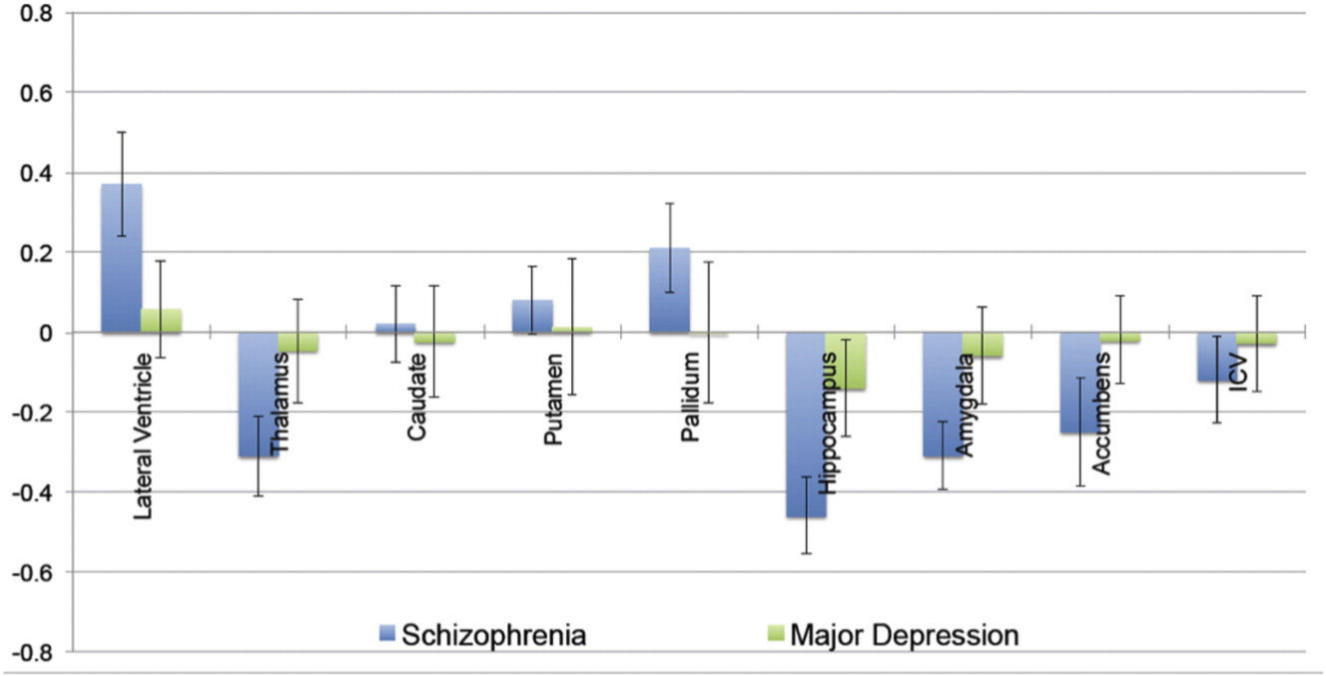
ENIGMA's studies of brain differences in disease revealed consistent patterns of subcortical volume differences across multiple cohorts with schizophrenia and major depression (data reproduced, with permission, from van Erp et al., 2015; Schmaal et al., 2015, *Molecular Psychiatry*). Here we show the effect sizes (Cohen's *d*), for the mean volume difference between patients and matched controls, for a range of brain structures measured from MRI. After meta-analysis of all cohorts, in schizophrenia, a range of subcortical structures showed volumetric differences, including hypertrophy, which may be due in part to antipsychotic treatment. In major depression, the hippocampus is smaller in the depressed groups. Such data, for these and other brain measures, is now being compiled and analyzed across 12 disorders in ENIGMA (see Table 1 for a summary), and may be useful for classification, so long as relevant confounds, site effects, and co-morbidities are appropriately modeled and understood.

At the time of writing, ENIGMA's first studies of schizophrenia and major depression have been published; results are compared in Fig. 4. Some caveats are needed in showing these data side by side: the schizophrenia and major depression patients were not ascertained at the same sites, so site or geographic effects may be present.

Among the subcortical structures so far assessed, the hippocampus shows the greatest differences in each disorder in terms of statistical effect sizes — but in major depression, it is the only structure showing differences, of those assessed so far (Schmaal et al., 2015). Many other structures show volume deficits or even hypertrophy in schizophrenia; basal ganglia enlargement has been widely noted in prior studies of patients taking second-generation antipsychotics. In people with schizophrenia, abnormal ventricular enlargement has long been reported (as far back as Johnstone et al., 1976), but the natural variations in ventricular size make the effect size smaller for this structure, even though the absolute volume difference, on average, is greater than for other structures assessed. In major depression, the hippocampal volume difference was greater in patients who experienced more depressive episodes, and in those diagnosed before the age of 21 years, which were at least partly independent effects. This is in line with many prior reports of greater brain differences in those with an earlier onset of the disease. Studies of cortical measures are now underway across all ENIGMA disease working groups; many cortical regions are commonly implicated in psychiatric illness, so these analyses may offer a more complete picture relating brain structural differences to clinical measures, medications, and outcomes. At the same time, diffusion imaging studies are also underway; initial reports reveal consistent deficits in fractional anisotropy – a measure of white matter microstructure – for major white matter tracts in schizophrenia (Bora et al., 2011; Holleran et al., 2014; Ellison-Wright et al., 2014; Kelly et al., 2015); an interesting question is whether antipsychotic medications affect white matter (Ahmed et al., 2015) and brain connectivity (O'Donoghue et al., 2015) in a way that fits with their known effects on structural anatomy.

### Extensions and Refinements

Because of the worldwide scope of the ENIGMA studies, only the brain measures that were most readily measured have so far been examined. Clearly, there are measures that may be more relevant to each disease or closer to the action of disease-causing genes, but if they are difficult to harmonize and measure in a standard way, the available sample sizes will lag behind those available for the simpler measures. Because of decades of work on shape analysis of anatomy, several of the ENIGMA disease groups have begun to analyze and meta-analyze subcortical shape (Gutman et al., 2015a,b,c), to map the profile of volumetric effects with more spatial precision. These efforts will also determine whether shape metrics offer additional predictive value over and above standard metrics, and in which situations.

The ENIGMA-Laterality group is studying global trends in the profile of left–right differences in brain structure, and whether they relate to handedness, sex, and disease status, in over 15,000 people (Guadalupe et al., 2015, submitted for publication). Reduced or abnormal brain asymmetry has been reported in many brain disorders (Okada et al., in press), but the scope and generality of these differences is not yet understood. Also, many important aspects of human brain function show lateralization in terms of the underlying processing networks, but the biology of this specialization is poorly understood, as are factors that influence it. Whether brain asymmetry measures add value as diagnostic predictors, will be testable across ENIGMA.

ENIGMA-EEG is studying the influence of genetic variants on brain functional activity measured with scalp recorded electrical signals, in a combined dataset from 10,155 individuals, ranging from 5 to 74 years of age. EEG metrics of brain function mature rapidly with age, and relate to aspects of cognition such as the brain's processing efficiency; they also show abnormalities across many neurodevelopmental and psychiatric disorders. Combining data from several large twin and family datasets, the ENIGMA-EEG working group is performing a genome-wide association analysis of brain oscillatory power – a highly heritable trait – before proceeding to in-depth analyses of lateralized activity, brain connectivity, and network properties.

### Brain-Wide Genome-Wide Association Studies

Voxel-based mapping methods are complementary to approaches that measure the volumes of specific regions of the brain, and they allow comprehensive and unbiased searches for effects of disease or genetic variations across the brain. “Brain-wide” genome-wide searches, or “voxelwise GWAS” (Shen et al., 2010; Stein et al., 2010a,b) can involve over a trillion statistical tests. However, once we account for the covariance within the image and genomic data, the number of independent tests being conducted drops to less than 15,000 × 1,000,000. Given the extremely low *p*-values of some genetic associations in ENIGMA (*p* ~ 10^–23^ in Hibar and the CHARGE and ENIGMA2 Consortia, submitted for publication; Hibar et al., 2015a,b, in press), several effects can still survive a “double” Bonferroni correction for multiple testing across both the image and the genome (Medland et al., 2014).

**Fig. 5:**
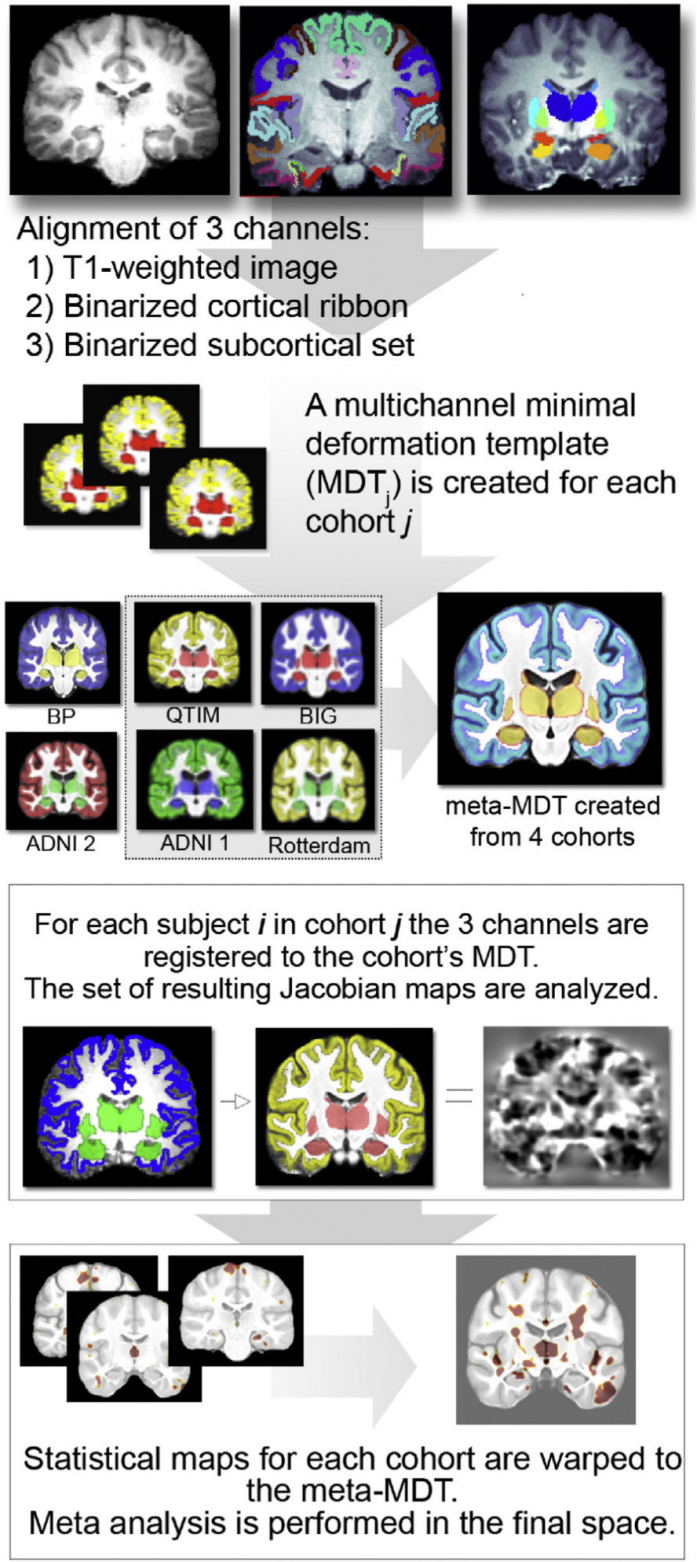
Meta-Analyzing Statistical Brain Maps As in other fields of brain mapping, voxel-based statistical analyses can map statistical associations between predictors and brain signals. To meta-analyze maps of statistical associations across sites, Jahanshad et al. (2015a,b,c) proposed a method whereby each site aligns data to their own brain template (mean deformation template, or MDT). Statistics from each site are meta-analyzed at each voxel, after a second round of registration to an overall mean template (computed here from 4 cohorts representing different parts of the lifespan). Analyses proceed in parallel, using computational resources across all sites; analyses are updated when a new site joins. This approach applies equally to voxel-based maps of function, and the ENIGMA-Shape working group has modified it to work with surface-based coordinates (Gutman et al., 2015a,b,c). If structural labels are used to drive the multi-channel registration (*top panels*), in conjunction with an approach such as tensor-based morphometry, the resulting local volumetric measures should closely mirror volumetric findings for specific regions of interest. As such, some results of brain-wide genome-wide searches can be checked by consulting genome-wide association results for specific regions of interest (Hibar et al., 2015a,b; Adams and the CHARGE and ENIGMA2 Consortia, submitted for publication).

As a result, several recent approaches have been developed to perform brain-wide genome-wide association studies to identify “spatial” features associated with genetic variants, such as specific WM pathways and their components, patterns of cortical thickness, or even activation patterns, rather than “global” measures such as brain or subcortical structure volumes. These approaches may be broadly divided into (1) *“brute force”* methods, that use mass-univariate testing to test every SNP for associations at each voxel in the image, and (2) *data reduction* methods, that attempt to reduce the search space by reducing the number of features in the image, or the genome, or both (Vounou et al., 2010, 2012; Ge et al., 2012). Data reduction methods may include classical methods, such as canonical covariates analysis, or independent components analysis (Gupta et al., 2015; Calhoun et al., 2015), or modern variants such as sparse coding, compressive sensing, or “deep learning” for feature discovery (see Thompson et al. (2013) for a review of multivariate imaging genomics methods). Among the “brute force” methods, Jahanshad et al. (2015a,b) detail a practical method whereby several sites run a voxel-based morphometric analysis independently, using a GWAS or other covariate-based analysis at each voxel, and later communicate their findings to a central site for meta-analysis (see Fig. 5). This approach was able to map out in the brain and meta-analyze the effects of the top SNP from the ENIGMA2 study, which screened the genome for variants associated with the size of subcortical structures (Hibar and the CHARGE and ENIGMA2 Consortia, submitted for publication; Hibar et al., 2015a,b, in press). To avoid re-computing everything when a new site joins, this “meta-morphometry” approach allows cohorts to align their data to their own brain templates, which are later aligned to an overall mean template for meta-analysis. Such a distributed effort offers many advantages for imaging genomics, due to the vast number of predictors: as new cohorts join, each site's computational hardware can be leveraged by all the others. Such an approach allows cooperative computation on data without requiring all the data to be shared or ever transferred. This is an interesting area of cooperative machine learning that can also increase “buy-in” — opening up participation to countries with stricter data transfer laws.

As part of ENIGMA3, a genome-wide screen of the cortex, one subproject will adopt “genetic clustering” methods to identify coherent patterns of gene effects in the brain (Chen et al., 2013, 2015). Based on the notion of genetic correlation, brain regions or sets of voxels can be grouped into clusters with similar genetic determination. The standard decomposition of the brain into regions may be adapted to include genetic clusters, or new regions where genome-wide association may be more efficient (Chiang et al., 2012). This approach has already been applied to create genetic partitions of the cortex; initial work in ENIGMA will overlay pre-made partitions on the cortical data from each site. Genetic correlations can now be computed rapidly from GWAS summary statistics (B. Bulik-Sullivan et al., 2015; B.K. Bulik-Sullivan et al., 2015) making it feasible to compute and perform clustering on matrices of “genetic connectivity” whose entries are genetic correlations. The ENIGMA-GCTA Working Group is currently studying these methods, in multisite data.

Many disorders affect the brain's white matter and connectivity. Using diffusion tensor imaging (DTI), ENIGMA's disease working groups have begun to compile evidence across cohorts for differences in a range of DTI measures, which reflect white matter integrity and microstructure (Kelly et al., 2015). Several years of work went into harmonizing ENIGMA's DTI analysis protocols, to study which metrics are consistently heritable and reproducible across multiple twin and family cohorts worldwide (Jahanshad et al., 2013a,b; Kochunov 2014; Kochunov et al., 2015). These DTI protocols have been carried forward into ongoing GWAS and disease studies, and initial genome-wide screens of the structural connectome (Jahanshad et al., 2013a,b; de Reus et al., 2015). On the genetic side, ENIGMA working groups have also formed to assess other kinds of genetic variation, including copy number variants (CNVs), where abnormalities have been reported in autism, schizophrenia, and learning disabilities. The ENIGMA CNV helpdesk is now supervising supervising an initial analysis of CNV data in 13,057 people from 24 cohorts worldwide, after developing harmonized protocols for CNV “calling” and quality control. Participating cohorts include groups from Japan, Mexican-Americans, and people of Western European, Nordic or Swedish ancestry. Initial efforts are evaluating known “psychiatric” CNVs as predictors of MRI and DTI phenotypes computed in other ENIGMA projects. Challenges include the pooling of data from genotyping chips with different coverage; some have sparse coverage of SNPs in regions with segmental duplications or complex CNVs.

In a complementary initiative, the ENIGMA-Epigenetics working group is studying epigenetic processes such as methylation, which is an index of biological aging and lifecourse ‘stress’ that may explain an important proportion of the gene-environment contribution to expression of many common diseases such as stroke and dementia. The group is now performing epigenome-wide association studies (EWAS), across 14 cohorts from Asia, Australia, North America, and Western Europe, to test associations between DNA methylation and brain measures, initially focusing on total brain volume, subcortical volumes and cortical thickness and surface areas. The working group is analyzing methylation data from 9,000 people, of whom 5,000 have both methylation data and MRI. In addition, the ENIGMA-Epigenetics group is prioritizing the analysis of DNA methylation sites based on their effects on gene expression or association with stress- and anxiety-related phenotypes. There is some evidence of early life changes in stress response genes through methylation (Backhouse et al., 2015), just as early life events influence later life disease expression — notably stroke, white matter hyperintensities, and cognitive impairment. Of great interest are epigenetic changes throughout the life span, and with aging, which may predict mortality from all causes, as well as physical and cognitive performance. Associations are being tested first for brain phenotypes that are known to change the most across the lifespan, based on incoming information from ENIGMA's Lifespan study in over 10,000 individuals (Dima et al., 2015).

## Relevance to Individual Evaluation, and Longitudinal Assessment

ENIGMA was not designed to make predictions about individuals based on their scans and genomic data. As in most epidemiological studies, the power lies in aggregating so much individual data that subtle effects on the brain can be detected, including findings that each cohort's data were insufficient to detect. In other words, its primary goal has been to relate brain measures to disease and treatment effects, and to variants in the genome. With the aggregated data, it has been possible to determine how reproducible these patterns are worldwide. Also, for the study of treatment effects, ENIGMA does not have the ideal design. Ideally, one would prefer to have pre–post treatment longitudinal designs instead of the cross-sectional comparisons in ENIGMA, where medication status is often confounded by age, disease duration, comorbidity and disease severity.

Even if a large data sample is needed to discover a factor that influences the brain, it does not mean that it is irrelevant to individuals; *APOE* is one such example, discovered in 1993 by linkage analysis in pedigrees. More recently, a rare variant in the *TREM2* gene (Jonsson et al., 2013; Rajagopalan et al., 2013) was found to affect Alzheimer's disease risk and accelerate brain tissue loss as we age — perhaps doubling loss rates in old age and increasing AD risk by a factor of 2–4. This gene variant is undoubtedly important for those who carry it: it is found in a little under 1% of controls and a little over 1% of AD patients.

### How Does it Help to Predict Risk for Decline?

In current clinical practice, it is not recommended to notify a research participant of their *APOE* status, and most ethics boards clearly define the circumstances in which incidental findings or health-relevant information is communicated back to a research participant. In the case of *APOE*, participants are not typically informed of their genetic status, as there are no effective treatments for late–onset Alzheimer's disease. Still, discovering predictors of more rapid decline is useful for the pharmaceutical industry for understanding the behavior of participants in clinical trials, and can greatly improve drug trial design, reducing costs. *Enrichment* approaches use some characteristic of a patient to select them for a clinical trial — this may be prior response to a certain drug, or it also may be a prediction that they are more likely to decline (FDA, 2013). In the AD field, some clinical trials now select patients based on having a PiB-positive PET scan (Ikonomovic et al., 2008) – as evidence of incipient AD pathology – and the APOE4 risk genotype, as carriers are more likely to develop AD. This selective enrolment allows faster, less costly, and more well powered clinical trials, with demonstrable reductions in the number of patients needed to show treatment effects (Hua et al., submitted for publication).

ENIGMA's disease working groups are likely to broaden the set of known factors that help predict recovery or decline. In ENIGMA-HIV, for example, a key goal is to understand predictors of resilience — factors that might forecast healthy brain development after the use of antiretroviral treatment (Fouche et al., 2015). Crucially, it is important to know if a predictor of decline is specific to one cohort or likely to generalize to others, or if it is applicable in a limited set of situations. Understanding how *APOE4* and other major risk genes shift the lifetime trajectory of brain measures will also help determine how much they will help when used for clinical trial stratification. This is a goal of the ENIGMA-Lifespan group (Dima et al., 2015). Clearly, any predictors of suicidal behavior would be very important in the management and follow-up of patients with psychiatric disorders (Mathews et al., 2013), and a secondary project on suicidality was started within the ENIGMA-Depression working group (Rentería et al., submitted for publication). Similarly, factors that predict whether ADHD in a child will persist into adulthood, will have clinical utility (Hoogman et al., 2015). Ultimately, the stratification or clustering of ENIGMA cohort data into subtypes, based on imaging, clinical or behavioral data, may point to distinctions that help us understand the heterogeneity of these disorders. This heterogeneity, without models to disentangle it, makes individual patient predictions harder to make.

### Normative Data Across the Human Lifespan

One effort where ENIGMA may contribute to individual prediction and evaluation – albeit with some caveats – is the ENIGMA-Lifespan project (Dima et al., 2015). In this work, ENIGMA cohorts are invited to contribute volumetric measures from normal individuals in their samples, which span the age range from 2 to 92 years of age. Although some cohort studies focus on children or the elderly, many scan people across the lifespan, allowing the computation of age-trajectories for several key brain measures; the results show a remarkable difference in the maturational trajectory of different structures, supporting many earlier neurodevelopmental reports on the sequence of brain development (Gogtay et al., 2004; Sowell et al., 2004). To cope with the non-uniform sampling density of the cohorts, these overall trajectories must be interpreted cautiously; clearly some parts of the lifespan are better sampled than others, and unmodeled effects of scan site, demographics, and even cultural or environmental differences may drive some of the effects. Clearly, disentangling the driving factors is statistically complex, but the potential is there, to derive normative measures and models of our path through life, in cohort studies as diverse as ENIGMA. The life span analyses (and normative curves) are also highly relevant for neurodevelopmental disorders such as OCD, ADHD, autism, etc. — for early detection, and secondary prevention in at-risk populations. Eventually, there may even be efforts to train individuals in specific domains, to stimulate the maturation of specific brain areas that appear to be deviant from the norm curves.

Such normative data have possible applications for individual assessment, if used judiciously. In pediatrics, growth charts for height and weight offer metrics of where a child stands relative to others of the same age, as a *Z*-score for example. Similar metrics for brain structure, among others, may help in studies of neurodevelopment where interventions and treatments are used to promote healthy maturation, or recovery, as in the case of brain trauma, for example. Similarly, better trajectories to chart loss of brain volume with advancing age help in routine diagnosis of the individual with possible cognitive problems, by indicating first if their brain is within normal limits for age, and secondly the precise centile on which it lies (Farrell et al., 2009; Dickie et al., 2013, in press) – much more data is needed to populate these graphs, but (much like child growth charts) they have the potential to be highly valuable in routine clinical practice as well as research. Original scan data are being collected to expand these templates (e.g., www.brainsimagebank.ac.uk).

Norming of brain measures also has commercial applications (Ochs et al., 2015). ENIGMA relies heavily on developments in software for imaging and genotype acquisition, quality control, and analysis, that make standardized assessment possible. In some regions of the world, such as Thailand and Cambodia, ENIGMA has contributors who are interested in whether it makes sense to use brain development norms from Western cohorts, or build their own (Jahanshad et al., 2015a,b,c; Fouche et al., 2015). By comparing developmental trajectories across very diverse multi-cohort data, better answers to these and other practical questions are within reach.

## Machine Learning, Big Data, and Individual Prediction

With the advent of very large neuroimaging datasets, we can fit predictive models to the data and test them for their robustness. Our models of how diseases and genes affect the brain are constantly being tested and improved, especially in situations where statistical effects have previously been too small to discover, or have been confounded by factors that cannot be adjusted for. In GWAS for example, there are known genetic differences in allele frequencies across populations, and if these are not accurately modeled based on much larger datasets, and adjusted for using multidimensional scaling, they will confound the analysis and lead to spurious results - many more SNPs will show “effects” on the brain, ultimately turning out to be false positives. Years of “false alarms” (Farrell et al., 2015) led the genomics community to adopt strict standards for reporting effects, including a standard genome-wide significance threshold (described above). In addition, independent replication of effects is required. In imaging, a somewhat more flexible approach has been used, with approaches from FDR to random field theory and permutation all co-existing in the literature; the use of candidate brain regions or prior hypotheses in functional imaging studies is encouraged, but the use of candidate regions in genomics is sometimes hotly debated as leading to many false positive effects (Collins et al., 2012; Farrell et al., 2015; ENIGMA-DTI Working Group, 2014). Munafò and Kempton (2014) argued that the growing flexibility in analyses used in neuroimaging is increasing the reporting of false positive results, and meta-analyses may offer better estimates of the validity of claims regarding brain differences in major depression and bipolar illness, fields for which they meta-analyzed the neuroimaging literature.

Given the sample sizes attained, ENIGMA offers a framework not only for unrestricted searches, but also to test more focused hypotheses and provide internal replication using, for example, cross-validation methods. So far, the Working Groups have over 30 “secondary proposals”: many study clinical measures, disease subtypes, and patterns of behavior such as suicidality or negative symptoms, or other differences that might contribute to the heterogeneity of brain disease and outcomes. One such project, in the ENIGMA-Major Depression group, assesses the effects of childhood trauma on depression-related brain measures, a factor that may be modeled effectively by comparisons with data from the ENIGMA-PTSD group, where childhood trauma is also a major predictive factor. Partnerships between ENIGMA groups may resolve some sources of brain differences that are difficult to disentangle. In HIV+ people who abuse stimulant drugs, for example, white matter inflammation is commonly reported, while patterns of accelerated atrophy are often seen in HIV+ people who do not use intravenous drugs, especially in those carrying the *APOE4* genotype. These and other predictors can be assessed in partnerships between the ENIGMA-Addictions and ENIGMA-HIV groups, by determining a common core of predictor variables that can be harmonized.

More refined models are also needed: we now know that the profile and extent of brain differences in disease may depend critically on a patient's age, duration of illness and course of treatment, as well as adherence to the treatment, polypharmacy and other unmeasured factors. Differences in ancestral background, as determined based on genotype, are strongly related to systematic differences in brain shape (Bakken et al., 2011; Fan et al., 2015). Any realistic understanding of the brain imaging measures must take all these into account, as well as acknowledge the existence of causal factors perhaps not yet known or even imagined. The quest to identify individual predictors is therefore more likely to succeed in finding factors that affect aggregate risk and outcome in groups of individuals, rather than offer firm predictions regarding an individual.

A more immediately achievable goal, for ENIGMA, is to rank brain measures in terms of how well they do predict individual decline, or diagnosis. Predictors of imminent brain decline are already used to boost the power for clinical trials in Alzheimer's disease, by over-enrolling, or separately analyzing patients whose brain measures, or clinical and genomic measures, suggest that they will decline faster. In ENIGMA, the ENIGMA-Plasticity group is evaluating the genetic influences on measures of brain change, in a meta-analytic setting (Brouwer et al., 2015). If reproducible drivers of brain decline could be found by screening brain data worldwide, they would help in planning enrichment approaches for drug trials. Several major initiatives have this goal (e.g., ADNI; Jack et al., 2015). Currently, the only genetic marker used for enrichment is *APOE*, but this may change as more information accumulates (see Lupton et al., submitted for publication). The complex pattern of association between brain measures and SNPs across the *APOE* gene (Hibar and the CHARGE and ENIGMA2 Consortia, submitted for publication; Hibar et al., 2015a,b, in press) suggests that future polygenic predictors based on machine learning may better predict clinical decline, and decline in brain measures, than the standard *APOE* genetic test, which is based on just 2 SNPs.

### Machine Learning

Innovations in machine learning make it possible to build robust predictive models from millions of predictors, often using dimension reduction techniques to home in on more efficient sets of variables that explain the most variance in the data; this vast field, including sparse learning and compressive sensing, is especially valuable in imaging genomics, with millions of predictors in both the images and the genome. Several machine learning developments have been applied to connect genomic and imaging measures, using methods such as parallel ICA (Gupta et al., 2015; Calhoun et al., 2015), elastic net (Wan et al., 2011), sparse reduced rank regression (sRRR; Vounou et al., 2010), among others. ENIGMA is beginning to test some of these models, specifically in the disease working groups, for case-control differentiation and differential diagnosis. Past efforts to combine imaging and genomic data for outcome prediction suggest that imaging measures may be much more predictive of future clinical decline than genomic measures, but both are complementary (Peters and the Alzheimer's Disease DREAM Challenge, submitted for publication). Predictive models should improve as they draw on more data, and the larger ENIGMA GWAS studies are now discovering more genetic markers that can be used in predictive models for brain measures (Hibar and the CHARGE and ENIGMA2 Consortia, submitted for publication; Hibar et al., 2015a,b, in press; Adams and the CHARGE and ENIGMA2 Consortia, submitted for publication). However, compelling as these approaches are and not wishing to dampen the enthusiasm for these very promising techniques, the image measurements being predicted generally require a human check and correction if necessary, particularly in datasets with complex imaging features such as occur in older patients with stroke – machine learning analysis algorithms still cannot reliably separate the hyperintensity due to a small cortical infarct from that due to a white matter hyperintensity or artifact, reliably. Also, the variants driving the heritability of disease risk are only just beginning to be discovered for many of the major brain diseases studied within and outside of ENIGMA. Unsupervised learning is also relevant for understanding the heterogeneity of diseases, which has made it harder to discover their causes and mechanisms. Brodersen et al. (2013) argued that one could use unsupervised learning on imaging, clinical and genetic data to see whether subtypes (or clusters) can be identified within a disease, and whether these data cluster together in agreement (or disagreement) with current diagnostic classifications.

In conclusion, we have reviewed current work by the ENIGMA Consortium. ENIGMA began in 2009, and is now a distributed effort, with over 30 working groups (see Table 1), coordinated from many centers worldwide. As we noted, ENIGMA's main goals have been to detect effects of disease and genetic variants on the brain, to see how consistent these effects are worldwide, and to study what modulates these effects. On the genetic side, it may soon be possible for polygenic scoring to produce predictors that are routinely used in brain imaging studies, explaining some of the observed variance. This may make other effects easier to detect. On the disease side, we are beginning to identify and confirm distinctive patterns of brain differences in each of a range of brain diseases, along with a better understanding of which patterns are specific to given disorders, which patterns tend to generalize, and what factors account for the heterogeneity across cohorts. This will help us understand the situations where predictive models can be used, for diagnostic classification, outcome prediction, and norming of individual data against appropriate reference populations.

We end with a note in praise of small studies. Like any consortium, ENIGMA would be impossible without the cohort studies and all the individuals who contribute; most of the data analyzed in ENIGMA came from cohorts with relatively modest sample sizes. Inevitably, many hypotheses are not addressable on a large scale, and some questions - especially causal questions - involve targeted interventions or phenotypic assessments with a depth or sophistication not likely to be attained at every site. As Aristotle said, “Nobody has the ability to work everything out, but everyone has something useful to say; working together, the whole vast world of science is within our reach.” (ἐκ πάντων δὲ συναθροιζομένων γίγνεσθαί τι μέγεθος; Aristotle, *Metaphysics α*, c. 350 BCE). This is the ENIGMA motto: http://enigma.ini.usc.edu/about-2/.

## Figures and Tables

**Table 1:** ENIGMA working groups, showing the number of independent participating samples, and the total sample size analyzed to date. A range of recruitment methods are represented. Some ENIGMA working groups, such as ENIGMA-Lifespan, ask questions that can be answered in healthy cohorts – often participants are controls from psychiatric studies, or population based samples, in which people with a current psychiatric diagnosis may be excluded altogether. Members of ENIGMA disease working groups have contributed their controls to several ongoing studies, leading to normative samples of unprecedented size (over 10,000 in the Lifespan and 15,000 in the Lateralization groups). Some working groups study clinic-based samples of cases and controls, and others study samples enriched for certain risk factors: over half of the people enrolled in ADNI, for example, have mild cognitive impairment, which puts them at heightened risk for developing Alzheimer's disease. In ENIGMA-Lateralization, one participating cohort (BIL&GIN) enrolls left-handers at a higher frequency than found in the general population, to boost power to understand handedness effects. Study designs, enrolment and sampling approaches vary widely across cohorts taking part in ENIGMA, so several ENIGMA studies assess how much difference it makes to restrict or broaden analyses in certain ways, such as pooling or separating certain categories of patients. Genetic analyses, for example, are typically run twice, first including patients and then excluding them. Disease group analyses may assess brain differences in different patient subgroups – chronically ill versus first-episode patients, at-risk siblings versus the general population, or people with different symptom profiles, or with distinct etiologies (e.g., negative symptoms, whose origin may differ in schizophrenia, addiction, or PTSD).

ENIGMA working groups	Number of cohorts	Total N (patient N)	Age range (in years)	Relevant publication(s)
ENIGMA2 GWAS (Subcortical)	50	30,717 (3,277 patients)	8-97	Hibar +287 authors, Nature, Jan. 2015
ENIGMA3 GWAS	50 +	32,000+ (4,000 patients)	8-97	In progress
ENIGMA DTI GWAS	35	13,500 (3,000 patients)	neonates-90	(Kochunov et al., 2014, 2015 NIMG; Jahanshad et al., 2013a,b NIMG)
ENIGMA EEG	4	10,155 (1,000 patients)	5-74	In preparation
ENIGMA-CNV	24	13,057 (1,800 patients)	13-90	In preparation
ENIGMA-Epigenetics	14	9,000	Across the lifespan	In preparation
ENIGMA-Schizophrenia	26	7,308 (2,928 patients)	average dataset age ranges from 21 to 44	van Erp et al., 2015, Mol Psych.
ENIGMA-MDD (Major depression)	20	10,105 (2,148 patients)	12-100	Schmaal et al., 2015, Mol Psych.
ENIGMA-BPD (Bipolar disorder)	20	4,304 (1,710 patients)	16-81	Hibar et al., in press, Mol Psych.
ENIGMA-ADHD	23	3,242 (1,713 patients)	4-63	Hoogman et al., OHBM, 2015, under review Am J Psychiatry
ENIGMA-OCD	35	3,722 (1,935 patients)	6-65	In preparation
ENIGMA-Epilepsy	23	6,569 (3,800 patients)	18-55	In preparation
ENIGMA-PTSD	15	4,555 (1,050 patients)	8-67	In preparation
ENIGMA-Parkinson's	4	950 (626 Patients/SWEDD)	30-85	In preparation
ENIGMA-22q	22	1,020 (554 patients)	6-50	in preparation; Sun et al., SFN 2015 (abstract); Schneider et al., AJP, 2014; Vorstman et al., JAMA Psych, 2015
ENIGMA-ASD (Autism Spectrum Disorders)	20	1,960 (1,074 patients)	3-46	In preparation
ENIGMA-HIV	10	650 (all patients)	6-85	Fouche et al., OHBM, 2015; Nir et al., CNS, 2015
ENIGMA-Addictions	21	12,458 (3,820 patients)	7-68	Mackey et al., PBR, 2015
ENIGMA-GCTA	5	4,000+	14-97	In preparation

Secondary Projects	Number of cohorts	Total N	Age range (in years)	Relevant publication(s)
ENIGMA-Lifespan	91	10,672 (healthy only)	2-92	Dima et al., 2015
Psychiatric cross-disorders	87	21,199 for 4 of the disorders (7,294 patients) Schizophrenia: 4,568 (2,028 patients) Bipolar Disorder: 4,358 (1,745 patients) Major Depression: 9,031 (1,808 patients) ADHD: 3,242 (1,713 patients)	4-100	-
ENIGMA-Lateralization	48	15,531 (0 patients)	8-90	Guadalupe et al., OHBM, 2015, submitted for publication
ENIGMA-Plasticity	10	2,513 (2,153 healthy controls; 290 schizophrenia patients; 70 bipolar disorder patients)	9-73	Brouwer et al., OHBM, 2015
ENIGMA-vGWAS meta-analysis	7	6,000	21-90	Jahanshad et al., OHBM, 2015, MICCAI 2015
ENIGMA-Schizophrenia-DTI	16	4,180 (1,927 patients)	18-60	Kelly et al., OHBM, 2015
ENIGMA-Schizophrenia-Relatives	8	4,079 (1,769 controls, 906 schizophrenia patients, 1,404 relatives)	8-58	In preparation
ENIGMA-Schizophrenia-shape	2	462 (159 patients)	16-75	Gutman et al., OHBM, 2015; Gutman et al., ISBI, 2015
ENIGMA-ILAE polygenic risk collaboration	12	34,992 (8,835 patients)	18-70	Whelan et al., 2015
ENIGMA-MDD (Major depression) DTI	15	2,100 (800 patients)	12-100	In preparation
ENIGMA-PGC Schizophrenia Collaboration	PGC Schizophrenia and ENIGMA2 summary statistics	PGC-Schizophrenia GWAS was based on 36,989 patients and 113,075 controls	8-97	Franke et al., in press; Stein et al., 2015
ENIGMA-Connectome-Methods harmonization	3	127 (healthy only)	21-85	de Reus et al., 2015

**Abbreviations: SWEDD =** scans without evidence of dopaminergic deficit.

## Footnotes

2 The term “endophenotype” was coined by John and Lewis (1966); in psychiatric genetics, it is used to denote a biomarker that fulfills several criteria (Gottesman and Gould, 2003; Glahn et al., 2014), including heritability, reproducible measurement, segregation with illness in families and in the general population, and state-independence — it must remain stable when a patient's illness is active or in remission. Others used the term “intermediate phenotype” for the brain measures studied in imaging genetics, as the endophenotype refers to the characteristics that are shared by both patients and their unaffected first-degree family members.

3 Abbreviations: SYS, Saguenay Youth Study, http://www.saguenay-youth-study.org; EPIGEN, The Epilepsy Genetics (EPIGEN) Consortium (Cavalleri et al., 2007); ADNI, Alzheimer's Disease Neuroimaging Initiative (http://www.adni-info.org); IMAGEN, IMAging GENetics Consortium (http://www.imagen-europe.com).

4 Obviously the SNPs are “known” in the sense that they are on the genotyping chip. The issue is that we do not know exactly which specific sets of SNPs or genes are truly contributing to a trait.

5 Linkage disequilibrium is the presence of statistical associations between alleles (genomic variants) at different loci in the genome, which arise because nearby regions on the genome tend to be inherited together. Maps of the level of LD between adjacent SNPs on the genome have been compiled for multiple ethnic groups. In imaging, LD leads to peaks of association with brain measures, and these LD maps can be used analytically to estimate SNP-based measures of heritability or genetic correlations from GWAS summary statistics.
